# Fluid-structure interaction of a patient-specific abdominal aortic aneurysm treated with an endovascular stent-graft

**DOI:** 10.1186/1475-925X-8-24

**Published:** 2009-10-06

**Authors:** David S Molony, Anthony Callanan, Eamon G Kavanagh, Michael T Walsh, Tim M McGloughlin

**Affiliations:** 1 Centre for Applied Biomedical Engineering Research (CABER), Department of Mechanical and Aeronautical Engineering and Materials and Surface Science Institute, University of Limerick, Ireland; 2 Department of Vascular Surgery, Mid-Western Regional Hospital, Limerick, Ireland

## Abstract

**Background:**

Abdominal aortic aneurysms (AAA) are local dilatations of the infrarenal aorta. If left untreated they may rupture and lead to death. One form of treatment is the minimally invasive insertion of a stent-graft into the aneurysm. Despite this effective treatment aneurysms may occasionally continue to expand and this may eventually result in post-operative rupture of the aneurysm. Fluid-structure interaction (FSI) is a particularly useful tool for investigating aneurysm biomechanics as both the wall stresses and fluid forces can be examined.

**Methods:**

Pre-op, Post-op and Follow-up models were reconstructed from CT scans of a single patient and FSI simulations were performed on each model. The FSI approach involved coupling Abaqus and Fluent via a third-party software - MpCCI. Aneurysm wall stress and compliance were investigated as well as the drag force acting on the stent-graft.

**Results:**

Aneurysm wall stress was reduced from 0.38 MPa before surgery to a value of 0.03 MPa after insertion of the stent-graft. Higher stresses were seen in the aneurysm neck and iliac legs post-operatively. The compliance of the aneurysm was also reduced post-operatively. The peak Post-op axial drag force was found to be 4.85 N. This increased to 6.37 N in the Follow-up model.

**Conclusion:**

In a patient-specific case peak aneurysm wall stress was reduced by 92%. Such a reduction in aneurysm wall stress may lead to shrinkage of the aneurysm over time. Hence, post-operative stress patterns may help in determining the likelihood of aneurysm shrinkage post EVAR. Post-operative remodelling of the aneurysm may lead to increased drag forces.

## Background

Abdominal aortic aneurysm (AAA) is a localized disease of the abdominal aorta. This dilatation of the infrarenal aorta has been found to affect 8.9% of the population over age 65 [1]. If the aneurysm continues to expand it may eventually rupture. Currently the decision to operate is based solely on the diameter of the aneurysm. When the maximum diameter exceeds 55 mm or the expansion of the aneurysm is greater than 10 mm/year surgery is normally recommended. The traditional approach to treating this disease is termed open repair, which involves an incision in the abdomen and the exclusion of the diseased aneurysm with a synthetic graft.

An alternative treatment is endovascular aneurysm repair (EVAR). This is the minimally invasive technique of inserting a stent-graft into the aneurysm site via the femoral and iliac arteries. The function of the stent-graft is to shield the aneurysm from the systemic blood pressure. Complications associated with EVAR include graft migration and endoleak. The occurrence of these complications may result in continued expansion of the aneurysm and possible rupture after EVAR [2,3]. Clinically it has been shown that aneurysms can still expand after EVAR without the presence of endoleak [4]. This phenomenon has been termed "endotension". Although such complications have been shown to be relatively rare, ruptures have been reported [5].

Numerical modelling of aneurysms and stent-grafts is a useful method for determining the stresses and forces seen in-vivo. Finite element analysis (FEA) allows the stresses on the aneurysm wall to be determined [6,7]. This method has shown that peak wall stress is more commonly associated with aneurysm rupture than maximum diameter [6]. Computational fluid dynamics (CFD) allows for the investigation of flow patterns and drag forces [8]. Drag forces on stent-grafts, which may influence stent-graft migration, have been investigated by several authors [9-11].

Fluid-structure interaction (FSI) of aneurysms, though still an emerging field of study has seen an increasing number of publications in recent years and with improved computer power and software capability this is expected to grow. There have been several patient-specific FSI simulations of aneurysms. These have taken into account non-linear wall models [12], iliac bifurcations [13] and intraluminal thrombus (ILT) [14,15].

However, there has been little work published on the effect of a stent-graft on aneurysm wall stress. Furthermore, only a few authors have studied patient-specific cases, two of these were flow studies only [10,16], while another studied the effect of the stent-graft on the wall mechanics [17]. Thus, these studies ignored fluid structure interactions effects. Previous fluid-structure interaction in 3D representative models have ignored the presence of ILT and considered the sac to be composed of stagnant blood [18-20]. This assumption may be imprecise as it has been reported that 75% of all AAAs contain ILT [21]. Also, this work did not assess the impact of remodelling of the aneurysm over time. Nonetheless, these studies have shown that the stent-graft can reduce peak aneurysm wall stress 20 fold.

The objective of this work was to investigate the biomechanics in a Pre-op, Post-op and Follow-up patient-specific abdominal aortic aneurysm with the use of fluid-structure interaction. Non-linear material models were used for the aneurysm and thrombus and the resulting wall mechanics were calculated. Finally the drag force on the stent-graft was also determined.

## Methods

### Geometry

CT scan data from a single patient was obtained from the Midwestern Regional Hospital, Limerick in DICOM format. The study conformed to the Declaration of Helsinki, and was approved by the local research ethics committee. The patient gave written informed consent. The CT data consisted of a pre-op scan and a 6 month follow-up scan. Using Mimics 12.0 (Materialise, Belgium) 3D models were reconstructed from this data. Images were segmented from just below the infrarenal aorta to the bifurcation of the iliac arteries. The reconstruction technique has been described in previous work from our group [22].

**Figure 1:**
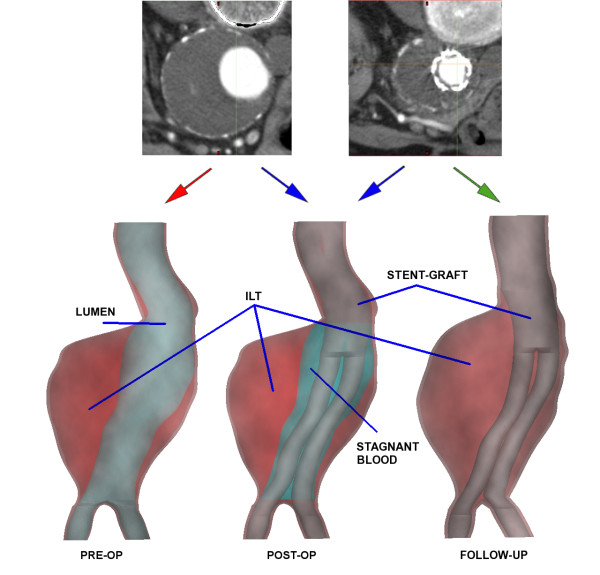
**3D model reconstruction**. The pre-op CT images are used to create the Pre-op model (red arrow), a combination of the pre- and post-op images are used to create the Post-op model (blue arrows) and the post-op images are used to create the Follow-up model (green arrow)

Three models in total were created, and will be termed Pre-op, Post-op and Follow-up from here on (figure 1). The pre-operative scan was used to identify the aneurysm and ILT from which the Pre-op model is created. The stent-graft lumen and stents can be identified in the post-operative scan. It was noted from the follow-up CT data that thrombus had begun to form in the stent-graft lumen, this was ignored in the reconstructions. The Follow-up model is created from this scan. Finally, the Post-op model was created from a combination of the pre-op and post-op scans. The follow-up stent-graft geometry was imported into the pre-op geometry and the graft was placed inside the aneurysm. The space between the ILT and the stent-graft was assumed to consist of stagnant blood. The aneurysm was saccular in shape and the geometrical properties are shown in table 1.

**Table 1:** Geometrical properties of patient

**Property**	**Pre-op**	**Follow-up**
Gender	Male	
Age	76	
V_AAA _(mm^3^)	264275	249633
V_ILT _(mm^3^)	185955	214166
% ILT	70	86
D_max _(cm)	7.6	7.19
D_in _(cm)	2.48	2.72
L_AAA _(cm)	9.6	9.9
L_AAA_/D_max_	1.26	1.37

Data relating to the patient and the geometry of the aneurysm.

Due to difficulties encountered during smoothing of the model in Mimics and also in order to achieve a tight contact between the stent-graft and aneurysm surfaces, the geometry in the form of polylines was exported as an iges file to Pro/Engineer (PTC, NH). Next, surfaces were created along the polylines and the outer aneurysm surface was offset 1.5 mm in order to give the aneurysm a suitable wall thickness. These surfaces were then exported to Gambit (Ansys, Canonsburg, PA) for meshing. Here both the fluid (lumen) and structure (aneurysm wall, thrombus and graft wall) domains are meshed. The meshes are then imported to Fluent 6.3.26 (Ansys, Canonsburg, PA) where the solid region can be exported to Abaqus 6.7-1 (Abaqus Inc, Pawtucket, RI).

### FSI

FSI is normally achieved either through a monolithic (full coupling) or partitioned approach (loose coupling) [23]. The commercial software MpCCI 3.0.6 (Fraunhofer SCAI, Germany) was used in this work. The software is based on the loose coupling of two chosen softwares. This approach allows the use of familiar and mature solvers for each domain. The benefits of using mature solvers are advanced capabilities that may not be available in monolithic solvers such as non-linear material models, contact and non-Newtonian fluid models.

For this research Abaqus, for the structural component, and Fluent, as the fluid solver, were coupled. The fluid time step was set to 0.001 s and data exchange occurred every 0.005 s, with Fluent sending the pressure to Abaqus, and Abaqus sending the deformed nodal co-ordinates to Fluent. Fluent uses the Arbitrary Lagrangian Eulerian (ALE) method to deal with the deforming mesh. A remeshing technique is used where cells are remeshed based on whether they violate a user specified size and skewness criteria. Specifically, the tetrahedral cells were remeshed if their size varied by more than 3% of the lower and upper size criteria. Both codes share a common boundary where the data exchange occurs. MpCCI identifies nodes or elements near each other based on an association scheme and data is then transferred from one node to the other. The software allows for non-matching meshes. Further information is available in the MpCCI documentation [24].

### Structural Model

The ILT and AAA wall were assumed to be hyperelastic, homogenous, incompressible and isotropic. The AAA wall and ILT were modelled using the constitutive model proposed by Raghavan and Vorp [25] and Wang et al. [26] respectively. The strain energy functions are given as:

(1)

(2)

where C_01 _= 0.174 MPa and C_02 _= 1.881 MPa are population mean values for the aneurysm wall; C_10 _= 0.026 MPa and C_20 _= 0.026 MPa are population mean values derived for the ILT. I_B _and II_B _are the first and second invariants of the left Cauchy-Green tensor **B **respectively. It has been shown that the use of population mean values does not significantly affect the prediction of stresses on the aortic wall [25]. The aneurysm and ILT were assigned values of 1120 kg/m^3 ^and 1121 kg/m^3 ^for the structural density respectively [27]. The stagnant blood in the aneurysm sac was assigned the same density as the lumen blood. The stent-graft was modelled as one whole body due to the difficulty in accurately reconstructing the nitinol stents from the CT data. A Young's Modulus of 10 MPa and a density of 6000 kg/m^3 ^were assumed for the stent-graft [27]. The graft and artery were assumed to be tied together, simulating attachment of the stent-graft to the artery wall, thus ignoring the possibility of endoleaks and local dislodgement.

**Figure 2:**
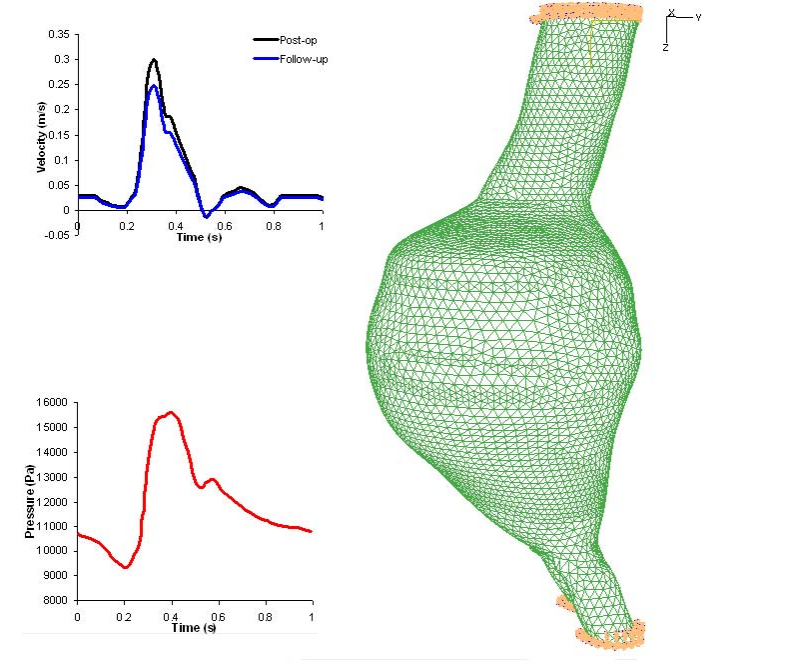
**Boundary conditions of Pre-op model**. (a) Inlet and outlet profiles and (b) solid mesh showing inlet and outlet constrained

The Pre-op and Post-op models had the same mesh for the aneurysm and thrombus. This consisted of 75,849 tetrahedral elements while the Post-op model had an additional 19,024 hexahedral elements for the stent-graft. The space between the ILT and stent-graft was modelled with 21,974 hydrostatic pressure elements in Abaqus. These simulated the stagnant blood in the aneurysm sac. The Follow-up model contained 26,368 hexahedral elements for the stent-graft and 64,350 tetrahedral elements for the aneurysm and ILT. For all models the aneurysm inlet and outlets were constrained in all degrees-of-freedom as shown in figure 2. This constraint, though non-physiological - as the inlet and outlets should be allowed to deform radially to correctly simulate tethering of the artery - is commonly assumed in finite element studies of aneurysms [7,28].

### Fluid Model

At the inlet a velocity boundary condition was assigned while at the outlets a pressure boundary was assigned (figure 2). The flow rate and blood pressure were not available for this patient so previously published data was used [29]. Peak systolic flow occurred at 0.305 s. The peak systolic pressure occurred at 0.4 s. Blood was assumed to be a Newtonian fluid with a density of 1050 kg/m^3 ^and a viscosity of 0.0035 Pas [16]. The PISO (Pressure Implicit with Splitting of Operators) algorithm was used for pressure-velocity coupling and a 2^nd ^order upwind scheme for discretization of each control volume. The pre-operative lumen consisted of 231,000 tetrahedral elements, while the post-operative and follow-up lumen (stent-graft) meshes contained 141,000 and 133,000 tetrahedral elements respectively.

### Mesh independence

Mesh independence was carried out separately on the solid and fluid meshes. The solid and fluid meshes were declared independent when the peak stress and wall force integral did not change by more than ± 2% between successive meshes. Pulse cycle independence was achieved after four cardiac cycles. CFD convergence criteria for mass and momentum residuals were 1 × 10^-4 ^and 1 × 10^-5 ^respectively. Simulations were performed on a 64 bit Dell Precision T7400 (2.99 GHz with 16 GB RAM) using 2 processors and one cardiac cycle took 40 hours.

In order to achieve further confidence in the accuracy of the results comparisons were made to clinical observations. Vorp et al. [30] found a compliance value of 4 × 10^-4^/mmHg in a clinical study of aneurysms. A similar value of 3.6 × 10^-4^/mmHg was seen in our study for the Pre-op model. Clinical investigations have shown reduced wall motion in aneurysms after EVAR Malina et al. [31] noted that the pulsatile wall motion was reduced by 75% after EVAR. Results in this study followed a similar trend with a reduction in compliance of between 80 and 96%.

## Results

### Wall Stress

**Figure 3:**
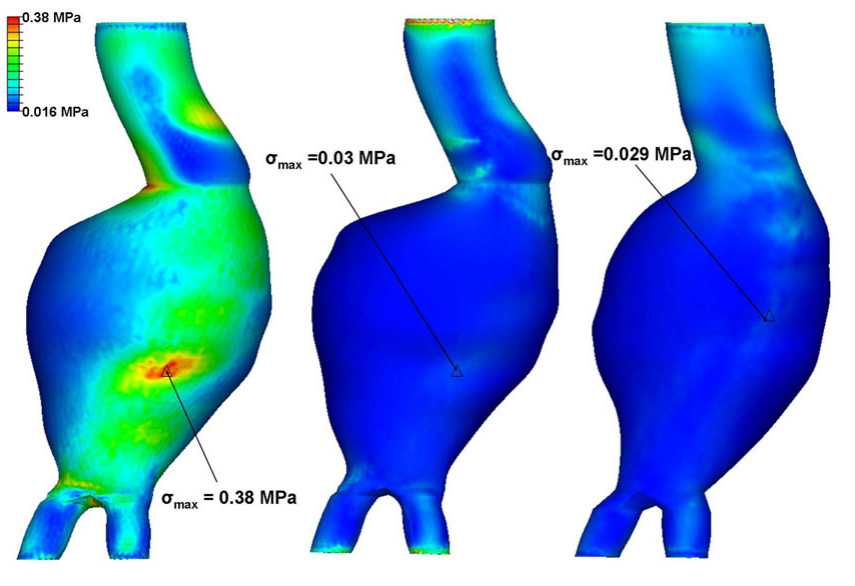
**Von Mises stress of Pre-op, Post-op and Follow-up models at time of maximum wall stress (t = 0.43)**. Pre-op model (left), Post-op model (centre) and Follow-up model (right) showing peak wall stress. Stresses are normalised to the pre-operative peak stress. The peak stress on the aneurysm sac region is indicated by the black triangle.

**Figure 4:**
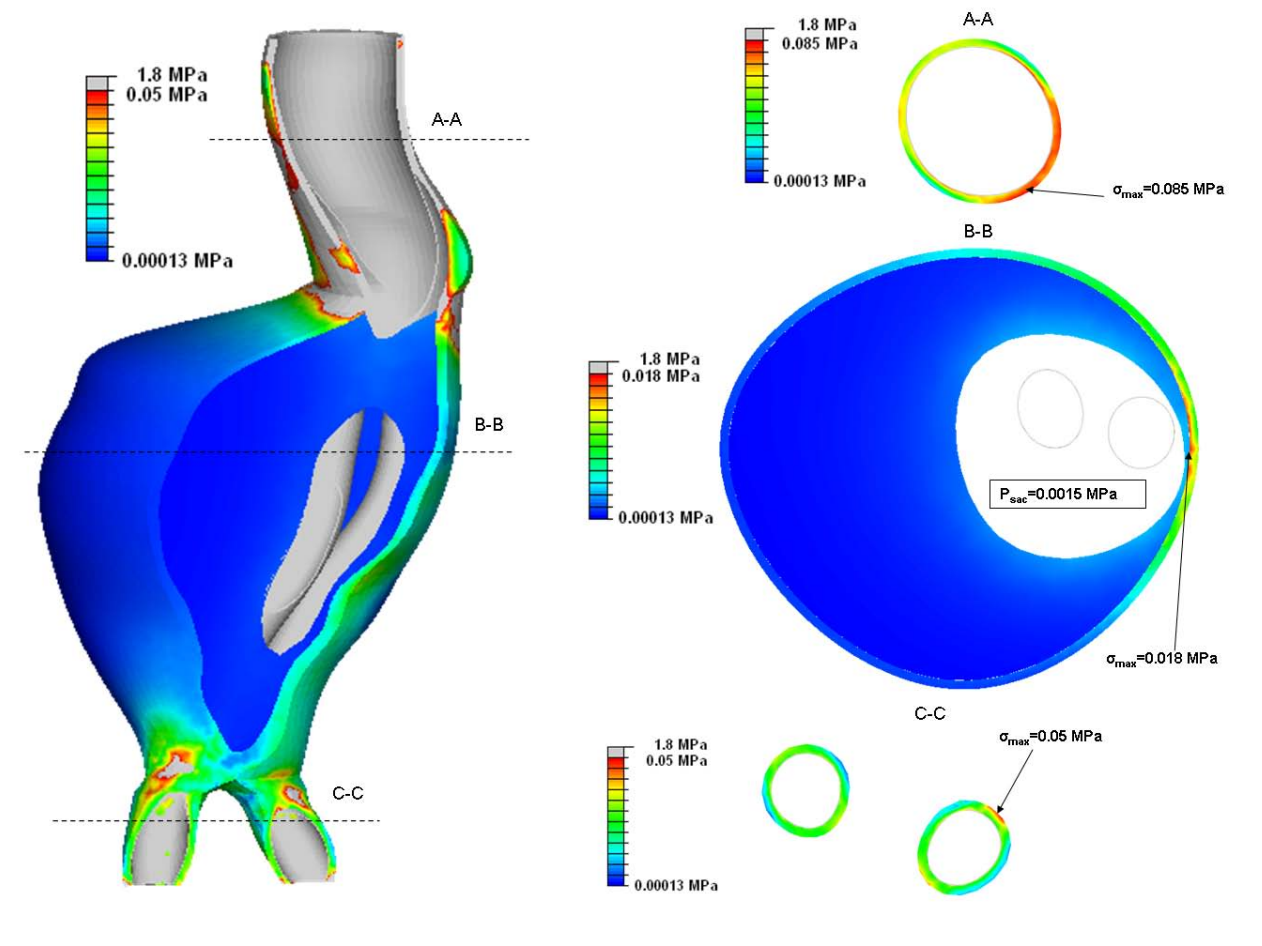
**Cross-section slices of Post-op model (t = 0.43)**. Three cross-sectional slices are taken through the Post-op model at the time of maximum wall stress (t = 0.43). These are termed A-A, B-B and C-C which refer to the aneurysm neck, aneurysm max diameter and iliac legs respectively. Stresses are normalised to the maximum local aneurysm stress in each slice.

The von Mises wall stress on the Pre-op, Post-op and Follow-up models are shown in figure 3. Any artificially induced high local stresses such as at the proximal and distal ends were ignored. These high stresses are caused by the boundary conditions constraining the model at both ends. The Pre-op peak wall stress was found to be 0.38 MPa. This was reduced by 92% to 0.03 MPa in the Post-op case. Peak wall stress was 0.029 MPa in the Follow-up model. The peak wall stress occurred on the posterior wall in all three models. For the Post-op model the pressure of the stagnant blood in the aneurysm sac varied from 0.001 MPa to 0.0015 MPa over the course of the pulse. Cross sectional slices from the Post-op model are shown in figure 4 (t = 0.43). The highest stress in the aneurysm is seen in the neck region where the stent-graft is in contact with the aneurysm wall. The stress in the stent-graft is much greater than the stress in the aneurysm and this is illustrated by the grey values. In slice B-B the highest aneurysm wall stress is located at a region where a very thin layer of thrombus separates stent-graft and aneurysm wall.

**Figure 5:**
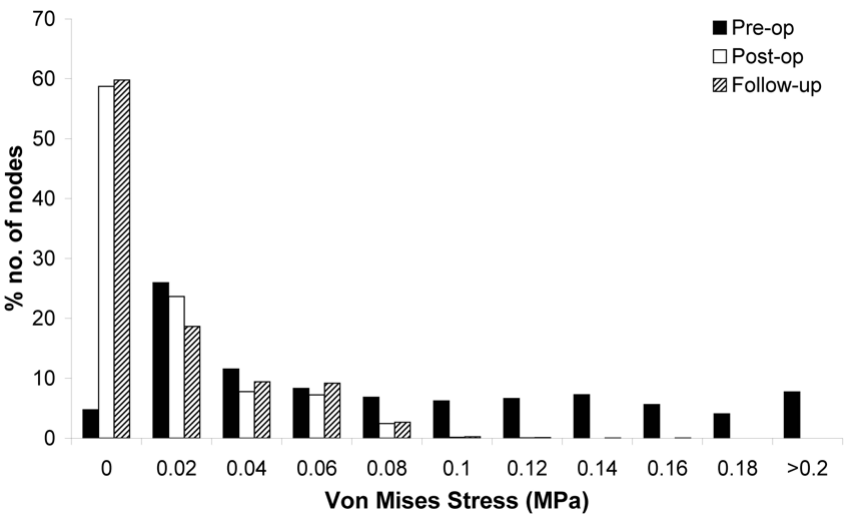
**Von Mises stress histogram**. Histogram showing the percentage distribution of stress in increments of 0.02 MPa for Pre-op, Post-op and Follow-up models.

**Figure 6:**
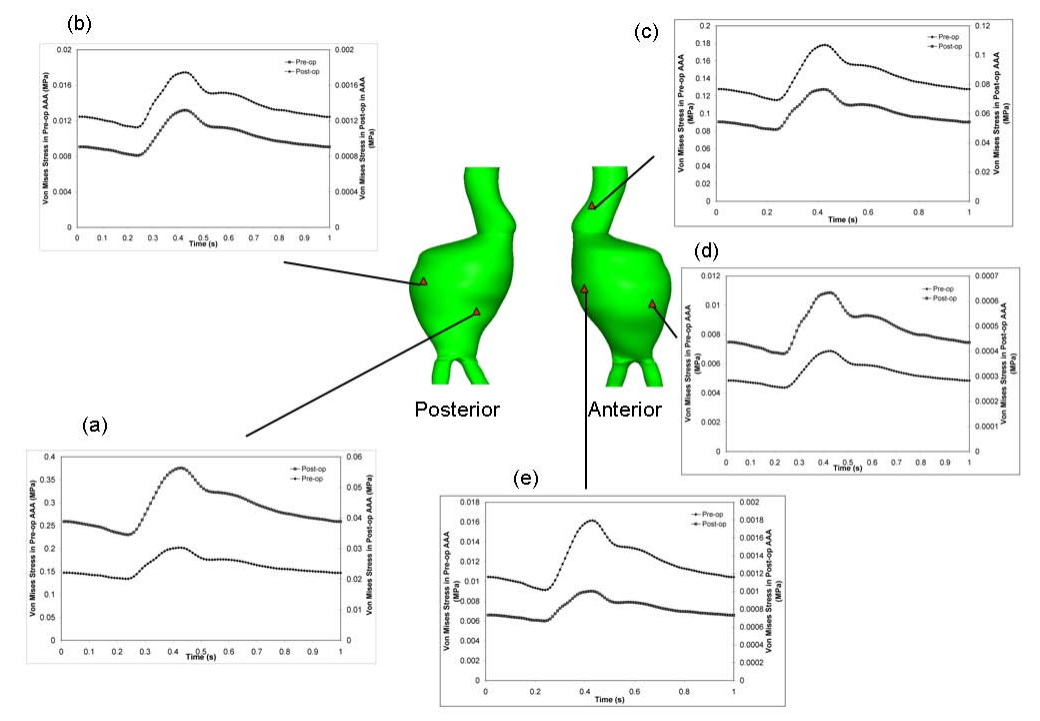
**Temporal von Mises stress distribution at 5 locations of interest on the aneurysm**. Temporal stress change in Pre-op and Post-op models at the (a) pre-op peak stress location, (b) maximum diameter on the posterior surface, (c) aneurysm neck, (d) post-op minimum stress location and (e) maximum diameter on the anterior surface. The left y-axis indicates pre-op stress and the right y-axis indicates post-op stress.

A histogram illustrates the effect of EVAR in redistributing the wall stress over the aneurysm volume (figure 5). Both the Post-op and Follow-up models had a greater no. of nodes with a lower von Mises stress than the Pre-op model. The temporal stress distribution was also measured at 5 different locations on the Pre-op and Post-op models (figure 6). The same node was chosen on each model for a direct comparison. In each location the stress was lower in the post-operative case. Similar levels of stress reduction were seen across the aneurysm sac, though the aneurysm neck had a much smaller reduction (table 2). For instance, at the pre-operative peak stress location the stress reduction was 92% while at the aneurysm neck, the stress reduction was 57%.

**Table 2:** Pre-op and post-op von Mises stress

	**Location**				
	**(a)**	**(b)**	**(c)**	**(d)**	**(e)**
Pre-op (MPa)	0.38	0.013	0.178	0.0069	0.016
Post-op (MPa)	0.03	0.0017	0.076	0.0006	0.001
% Difference	92	87	57	91	94

Table detailing the Pre-op and Post-op difference in von Mises stress at the five locations described in figure 6

### Compliance

**Figure 7:**
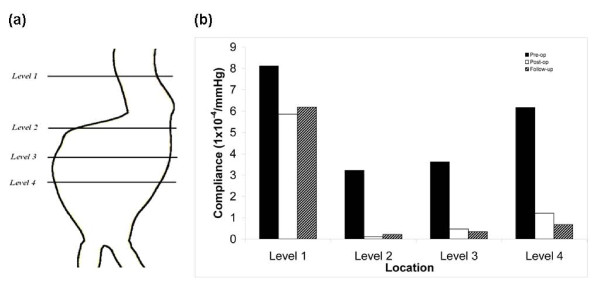
**Aneurysm compliance**. (a) Posterior view showing the locations of compliance measurements. Level 1 is at the aneurysm neck, level 2 is 20 mm above the maximum diameter, level 3 is at the maximum diameter and level 4 is 20 mm below the maximum diameter. Compliance measurements in the follow-up model are taken from the same axial co-ordinates (b) Chart showing compliance values for Pre-op, Post-op and Follow-up models

Compliance is a tool used to describe the distensibility of an artery. The compliance of the aneurysm (figure 7) was measured at four locations. Compliance is defined as [30]

(3)

where A_max _and A_min _are the measured area of interest corresponding to systolic pressure (P_max_) and diastolic pressure (P_min_) respectively. Compliance of the aneurysm was reduced post-operatively (figure 7). The difference in Pre-op and Post-op compliance was 27% in the aneurysm neck. This difference increased to 87% at the maximum diameter (table 3). A similar reduction in compliance occurred for the Follow-up model.

**Table 3:** Compliance measurements

	**Level 1**			**Level 2**			**Level 3**			**Level 4**		
	**Pre**	**Post**	**Follow**	**Pre**	**Post**	**Follow**	**Pre**	**Post**	**Follow**	**Pre**	**Post**	**Follow**
Systolic Area (mm^2^)	662	610	733	3877	3701	3298	4879	4657	4110	3789	3494	3495
Diastolic Area (mm^2^)	638	594	713	3821	3699	3295	4800	4648	4104	3684	3475	3476
ΔP (mmHg)	45			45			45			45		
% Difference in Compliance	27		23	96		93	87		90	80		89

Table detailing the Pre-op, Post-op and Follow-up differences in compliance. Systolic and diastolic areas were measured at four different locations along the aneurysm as shown in figure 7. ΔP is the difference between systolic and diastolic pressure. The % difference in compliance refers to the difference between Pre-op and Post-op and Pre-op and Follow-up.

### Velocity pathlines and pressure

**Figure 8:**
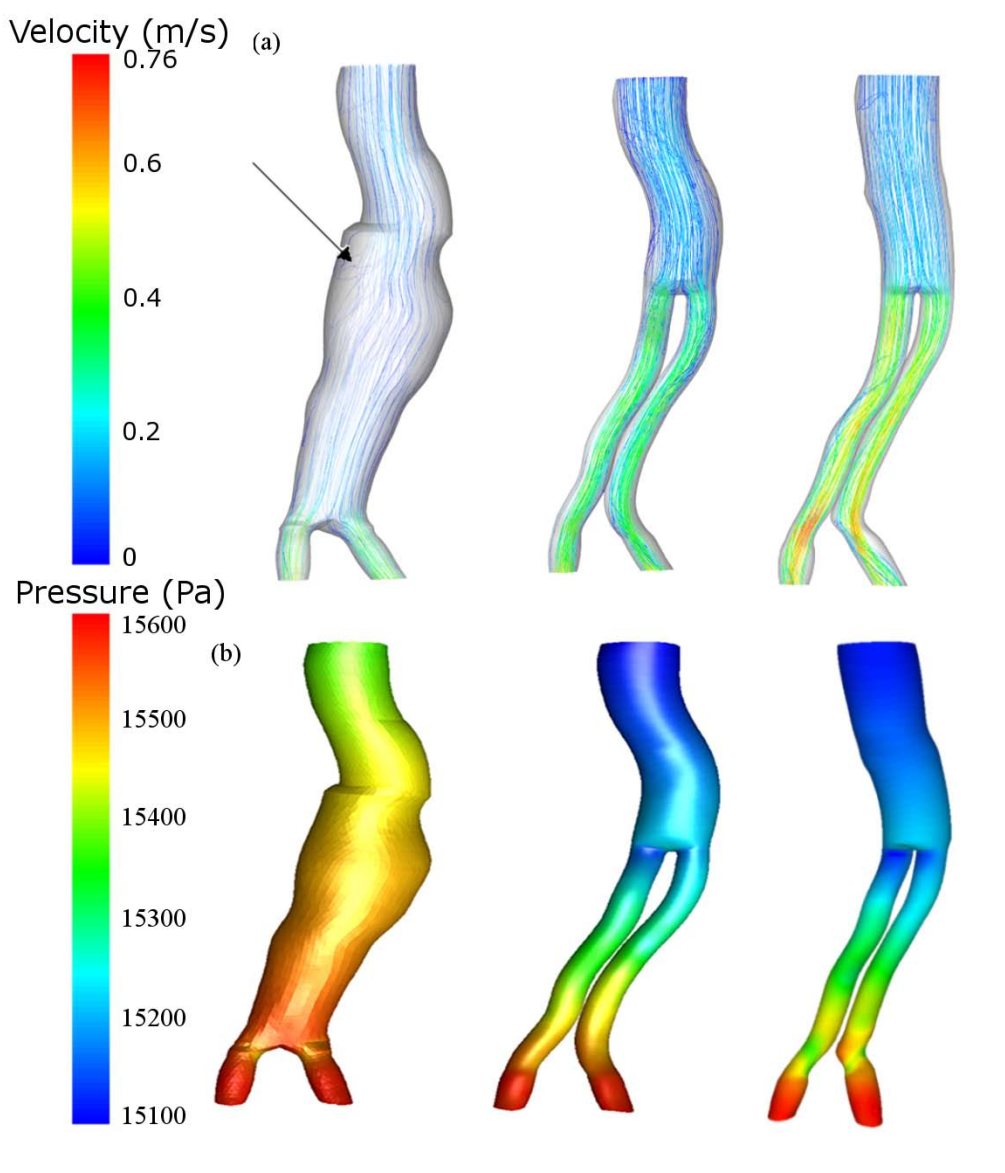
**Velocity pathlines and pressure**. (a) Velocity pathlines (m/s) for the Pre-op (left), Post-op (centre) and Follow-up (right) cases and (b) pressure contours (Pa) for the Pre-op (left), Post-op (centre) and Follow-up (right) cases. These results are taken at the time of peak pressure (t = 0.4). The arrow indicates the presence of a vortex.

During peak systolic pressure the flow of blood has begun to decelerate, which can lead to vortex formation [16]. The removal of a vortex after the graft has been implanted can be seen in figure 8. This occurs just after the blood flow leaves the aneurysm neck (indicated by arrow in figure 8). The velocity magnitude was greatest in the Follow-up model. A larger pressure gradient can be seen in the Post-op and Follow-up models than in the Pre-op model. This amounts to approximately a 250 Pa greater pressure drop in the stent-graft. The larger pressure gradient may be due to the greater curvature of the iliac limbs of the stent-graft.

### Drag Force

**Figure 9:**
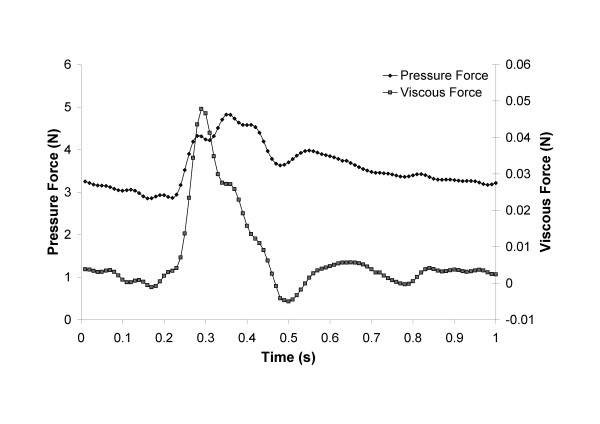
**Drag force**. The axial drag force acting on the post-op stent-graft over the course of the pulse. Both the pressure and viscous forces are shown.

The drag force is caused by pressure and viscous forces acting on the stent-graft [10]. Peak drag force in the Post-op and Follow-up models occurred prior to the peak systolic pressure with a value of 4.85 N and 6.37 N respectively. In the Post-op model the drag force varied from a value of 2.85 N to 4.85 N over the cardiac cycle, while in the Follow-up model the drag force varied between 3.75 N and 6.37 N. Both the viscous force and pressure force followed the same trend as the velocity and pressure waveform respectively (figure 9). The majority of the force was generated due to the pressure component. At peak systolic flow the viscous force was 1.1% of the total drag force, while at peak systolic pressure it was 0.35% of the total drag force.

### Influence of stent-graft Young's Modulus

In order to determine the influence of the stent-graft stiffness the Post-op simulations were performed again for a Young's Modulus of 5 MPa and 15 MPa (table 4). The peak aneurysm wall stress decreased for increasing graft stiffness. Similarly the compliance of the aneurysm decreased for an increase in graft stiffness. No significant changes were seen in the fluid flow. There was a small reduction in drag force for increasing graft stiffness.

**Table 4:** Effect of stent-graft Youngs Modulus

**Stent-graft Young's Modulus (MPa)**	**Peak drag force (N)**	**Peak aneurysm wall stress (MPa)**	**Compliance at proximal neck (1 × 10^-4^/mmHg)**	**Compliance at maximum diameter (1 × 10^-4^/mmHg)**
5	4.86	0.061	6.79	0.92
10	4.85	0.038	5.85	0.46
15	4.83	0.019	4.81	0.33

Table detailing the sensitivity of the results to a change in stent-graft stiffness.

## Discussion

Fluid-structure interaction of a Pre-op, Post-op and Follow-up AAA was simulated. The remodelling of the aneurysm over a 6 month period was taken into account. Previously reported parameters such as wall stress and drag force were examined as well as the additional parameter of compliance. Most previous work in this field has investigated numerous parameters in a representative model based on CT scans [18-20,27]. It was observed that peak wall stress is reduced 20 fold in a pre- and post-op AAA [18]. In a patient specific FEA model a 90% decrease in aneurysm wall stress was reported [17]. Our results compare favourably to both these studies. The peak von Mises wall stress on the aneurysm was reduced by 92% (12 fold) after EVAR in the Post-op model.

In the Pre-op case higher stresses are seen in the aneurysm region than in the neck region. The opposite is seen in the Post-op and Follow-up cases. This could be seen as an indication of the success of EVAR i.e. low post-operative stress on the aneurysm sac wall. The high stress on the aneurysm neck is due to the contact of the stent-graft with the aneurysm wall. The higher stress here may cause the aneurysm neck to expand which can lead to an increase in drag force [10]. From the Follow-up model it was noted that the proximal neck diameter increased from 24.8 mm to 27.2 mm. Neck enlargement after EVAR has been reported extensively [32]. Arterial remodelling occurs when local wall stress deviates from a reference value [33]. After EVAR there is normally a reduction in aneurysm volume and diameter [34]. Previous research has shown the peak wall stress in the normal abdominal aorta to be in the region of 0.225 MPa [35]. As can be seen in (figure 6a, b and 6d) the Post-op wall stress in the aneurysm sac region is below the normal aorta peak stress. The lower stress in this region may cause the aneurysm to shrink. The Post-op wall stress in the aneurysm neck is much closer to the stress seen in the normal aorta (figure 6(c)).

Qualitatively, the wall stress in the Post-op and Follow-up models were very similar (figure 3). The stress histogram also showed little difference between both models. The aneurysm volume was reduced by 15,000 mm^3 ^in the 6 months after EVAR. Despite this, in both cases approximately 60% of the nodes had a wall stress of less than 0.2 MPa. There did appear to be a greater number of nodes with high wall stress in the follow-up case which may reflect the reduction in aneurysm volume.

The incorporation of the ILT played an essential role in the stress distribution across the model. The area between the stent-graft and aneurysm bulge is encased by a large volume of thrombus (figure 4). The ILT transfers a reduced load to the aneurysm wall. In slice B-B the ILT has the largest influence on the stress distribution. The peak stress here - 0.018 MPa - occurs where a thin layer of ILT separates the aneurysm wall and stent-graft, whereas, on the opposite wall of the aneurysm the peak stress is 0.001 MPa. The thick layer of ILT reduces the stress on the bulge of the aneurysm. The role of ILT had been neglected in previous FSI studies of post-op AAAs [18-20]. Previous authors have also indicated the need to include ILT to obtain accurate stress results [22,26].

The compliance of the aneurysm refers to the compliance of the overall post-operative environment, which is dependent on the stent-graft, ILT, stagnant blood and aneurysm. If the aneurysm alone was to be considered, the elasticity and hence compliance have not actually altered from the pre-operative case. The only alteration has been to the overall environment, with the resulting reduction in compliance. An alternative method would have been to use the sac pressure but this would not have allowed us to make a direct comparison between the pre-op, post-op and follow-up models. Due to the ILT being a solid entity, there is no sac pressure in the follow-up model. Furthermore, clinically, intra-sac pressure measurements are not routinely recorded. The percentage difference between Pre-op and Post-op compliance was greatest at levels 2-4 (table 2). At level 1 the difference was much smaller. At this location the stent-graft pushes on the aneurysm neck causing it to deform, resulting in a post-op compliance closer to the pre-op compliance. There was little difference in compliance between the Post-op and Follow-up models. The overall reduction in aneurysm volume in the Follow-up case is not reflected by any significant alterations in aneurysm compliance.

The shape of the pre-op lumen and post-op stent-graft are quite similar. This is due to the large volume of ILT narrowing the pre-op lumen. Despite this similarity the stent-graft had smoother pathlines and noticeably in one region vortices were removed after the implantation of the stent-graft. Greater flow disturbances are likely in pre-op patients with less ILT, as when the lumen expands into the aneurysm sac vortices will develop in the pockets created. In the Follow-up model, the proximal neck has straightened due to remodelling and this may further improve the flow of blood.

As has been seen in previous studies the vast majority of the drag force magnitude is due to the pressure component [11]. The downward axial force is more likely to be associated with caudal graft migration than forces in the radial direction. The maximum axial drag force for the Post-op model was 4.85 N. The drag force increased in the Follow-up model to 6.37 N. This may be attributed to the greater neck diameter of the follow-up aneurysm, as it has been shown that grafts with greater proximal neck diameters result in a larger drag force. Previously Morris et al. found that in similar sized grafts with neck diameters of 25-27 mm and without significant curvature the drag force was approximately 5.5-6.5 N [11]. Bench-top testing has shown that forces ranging from 4.5 N to 40 N can dislodge stent-grafts [36,37].

The stiffness of the stent-graft was found to have little influence on the fluid domain. A negligible change in drag force of less than 1% was noted, which suggests that modelling the stent-graft as rigid may be an acceptable assumption if just the fluid forces are of interest. A change in the stent-graft stiffness had a greater influence on the compliance and aneurysm wall stress. Stiffer stent-grafts will reduce the stress and deformation of the aneurysm wall.

### Limitations

There are several limitations to the current study. The Post-op model assumes that there was no immediate remodelling of the pre-operative aneurysm due to the implantation of the stent-graft. The patient was selected from our patient database as the aneurysm geometry had not changed greatly over the 6 month follow-up period. Despite this, there was some remodelling of the aneurysm as can be seen by the straightening of the aneurysm neck and the formation of thrombus in the stent-graft lumen (figure 1). The main reason for creating the Post-op model was in order to make a direct comparison between the pre-op and post-op case. Exact nodal locations on the aneurysm could be compared as the same aneurysm mesh was used in the Pre-op and Post-op models. This was not possible in the Follow-up model due to the contrasting geometries and meshes and hence only the global characteristics can be compared.

Stent-grafts are oversized by 10-20% normally [3], but because the model is reconstructed from CT scans it is already in a state of stress so the unloaded configuration is unavailable. Hence, oversizing of the stent-graft was ignored. The stent-graft was modelled as a single body due to the difficulty in reconstructing the stents. Stents are radiopaque and so show up as high intensity pixels in DICOM images and can contain a lot of noise. This creates difficulties in reconstruction of the geometry as the stent-graft boundary can be difficult to identify. The wall thickness of the aneurysm could not be determined from the CT images. Furthermore, it was not possible to obtain the flow rate and blood pressure for the patient, as these are not routine measurements for AAA patients.

The tied contact between the stent-graft and AAA may not simulate the actual contact in-vivo. Stent-graft fixation is due to the radial force and friction exerted by the stent-graft on the aneurysm neck. A non-stick contact would have modelled this more appropriately but would also have significantly increased the simulation time. A loose coupling method was employed for the simulations, though in reality strong coupling exists between the fluid and solid in blood flow in arteries [23]. This is due to similar densities of the fluid and solid. A strong coupling method may better simulate the fluid-structure interactions. It has been shown through biaxial tensile testing that AAA behaves as an anisotropic material. The use of anisotropic properties has been shown to result in higher peak stresses than isotropic properties [28,38]. Similarly, calcifications in the aortic wall were neglected and these have been shown to result in higher peak stresses [39].

## Conclusion

To the best of the authors' knowledge this is the first fluid-structure interaction study of pre- and post-operative patient-specific AAA biomechanics. Aneurysm remodelling 6 months after implantation of the stent-graft was investigated. The peak wall stress on the aneurysm wall was reduced by 92% after EVAR. Compliance of the aneurysm is significantly reduced after EVAR. Aneurysm remodelling after EVAR may lead to an increase in stent-graft drag force. Hence, despite the success of EVAR in reducing aneurysm wall stress, patient follow-up is essential in order to determine the consequences of remodelling of the aneurysm.

## Abbreviations

AAA: Abdominal aortic aneurysm; CT: Computed tomography; FSI: Fluid-structure interaction; EVAR: Endovascular aneurysm repair; FEA: Finite Element Analysis; CFD: Computational Fluid Dynamics; ILT: Intraluminal thrombus; 3D: Three-dimensional; DICOM: Digital Imaging and Communications in Medicine; ALE: Arbitrary Lagrangian Eulerian; PISO: Pressure Implicit with Splitting of Operators.

## Competing interests

The authors declare that they have no competing interests.

## Authors' contributions

DSM reconstructed the models, conducted the simulations, analyzed the results and drafted the manuscript. AC analyzed the results and revised the manuscript. MTW analyzed the results, revised and gave final approval of the manuscript. EGK acquired and analyzed the CT data and revised the manuscript. TMM analyzed the results, revised and gave final approval of the manuscript. All authors read and approved the final manuscript.
